# Biofilm production and other virulence factors in *Streptococcus* spp*.* isolated from clinical cases of bovine mastitis in Poland

**DOI:** 10.1186/s12917-017-1322-y

**Published:** 2017-12-28

**Authors:** Edyta Kaczorek, Joanna Małaczewska, Roman Wójcik, Andrzej Krzysztof Siwicki

**Affiliations:** Department of Microbiology and Clinical Immunology, Faculty of Veterinary Medicine, University of Warmia and Mazury in Olsztyn, Oczapowskiego 13, 10-719 Olsztyn, Poland

**Keywords:** *Streptococcus*, Mastitis, Biofilm, Virulence genes

## Abstract

**Background:**

Mastitis is a common disease in dairy cattle throughout the world and causes considerable economic losses each year. An important aetiological agent of this disease is bacteria of the genus *Streptococcus*; hence, exploring the mechanisms of virulence in these bacteria is an extremely important step for the development of effective prevention programmes. The purpose of our study was to determine the ability to produce biofilm and the occurrence of selected invasiveness factors among bacteria of the genus *Streptococcus* isolated from cattle with the clinical form of mastitis in northeastern Poland.

**Results:**

Most of the isolates analysed demonstrated an ability to produce biofilm (over 70%). Virulence genes were searched for in the three most common streptococci in our experiment: *S. agalactiae, S. uberis* and *S. dysgalactiae*. For *S. agalactiae*, only four genes were confirmed: *rib* (33%), *cylE* (78%), *bca* (37%), and *cfb* (100%). The genes *pavA*, *scpB*, *bac* and *lmb* were not present in any of the tested strains. The dominant serotypes of the species were Ia (*n* = 8) and II (*n* = 8), in addition to some strains that were not classified in any of the groups (*n* = 6). Out of the eight selected genes for *S. uberis* (*sua*, *pauA/skc*, *gapC*, *cfu*, *lbp*, *hasA*, *hasB*, *hasC*), only one was not found (*lbp*). Finally, two genes were chosen for *S. dysgalactiae* (*eno* and *napr*), and their presence was confirmed in 76% and 86% of the strains, respectively.

**Conclusions:**

The experiment showed that strains of *Streptococcus* spp. isolated from dairy cattle with clinical cases of mastitis in the northeastern part of Poland possess several invasiveness factors that can substantially affect the course of the disease, and this should be considered when developing targeted prevention programmes.

## Background

Mastitis in dairy cattle is a globally widespread disease that is responsible for large economic losses each year due to lower milk yield and reduced milk quality [1]. One of the aetiological factors mentioned most often are bacteria of the genus *Streptococcus*, where pathogens classified as both contagious (e.g., *Streptococcus agalactiae)* and environmental (e.g., *Streptococcus uberis*) can be distinguished [2–4].

It is common knowledge that antibiotics are among the major tools in combating this disease [1]. However, to avoid development and spread of antibiotic resistance, the use of the antibiotics in the treatment of mastitis should be limited to situations where clinical and bacteriological findings justify their use. Sometimes, in spite of using an active substance that shows efficacy under in vitro conditions, antibiotic therapy is not effective. These situations are associated with the presence of various host and bacterial factors, including the ability of bacteria to produce biofilm [5, 6]. ‘Biofilm’ is defined as a matrix-enclosed microbial accretions which can adhere both to biological or non-biological surfaces. By producing various types of substances, the formation of biofilm is a significant factor in the pathogenesis of numerous diseases, both in humans and in animals [7–9].

By forming such a structure, bacteria are more likely to survive in an environment that is hostile to them, and after invading an organism, they can be better protected against the action of the host immune system while becoming less sensitive to the activity of antibiotics or disinfectants [7, 10]. In addition to the ability to produce biofilm, *Streptococcus* spp. possess other invasiveness factors, for example those that provide protection against the host immune system (e.g., plasminogen activator, lactoferrin binding protein, or CAMP factor) [11, 12]. For years, the presence of specific virulence factors in pathogens has drawn the attention of research teams throughout the world because such research increases our understanding of the mechanisms responsible for inducing an illness and can therefore contribute to the development of optimal prevention programmes.

To date, several studies have evaluated the ability of *Streptococcus* spp. to produce biofilms as well as the presence of virulence factors in bacteria isolated from dairy cattle suffering from mastitis in different countries; however, we are the first to examine such phenomena in our region [8, 11, 13]. Hence, the purpose of our study was to identify the ability of bacteria to make biofilm and to detect the presence of selected virulence factors in bacteria of the genus *Streptococcus* isolated from cattle with the clinical form of udder inflammation in northeastern Poland.

## Methods

### Sample collection

A total of 135 previously described streptococcal isolates were used in this study [14]. All samples were collected from 65 different farms in north-eastern Poland from 2013 to 2015. To avoid testing of epidemiologically related isolates, only 1 isolate from one species per dairy farm was included. Prior to milk sampling, all animals were tested using the California mastitis test. Only milk from quarters presenting symptoms of infection was collected (i.e., slight thickening of the mixture; trace reaction seems to disappear with continued rotation of the paddle, CMT ≥ 4). After sampling, milk was kept in a container that was maintained a constant temperature of 6 to 8 °C and was delivered to the laboratory within 2 h.

### Bacteriological identification

Milk samples were transferred with a calibrated loop (0.01 ml) onto Columbia agar (Oxoid, Great Britain) and Edwards medium (Oxoid, Great Britain), both supplemented with 5% defibrinated sheep blood. The plates were incubated at 37 °C for 48 ± 2 h under aerobic conditions. The grown cultures were examined microscopically after Gram staining, and the phenotypic traits were analysed (i.e., type of haemolysis, esculin hydrolysis, catalase production, CAMP reaction). For further analysis, only G+, catalase-negative cocci were selected. The final identification was performed using the commercial latex agglutination test Streptococcal Grouping Kit (Oxoid, Great Britain), API strep (bioMérieux, France) and PCR. All strains used in this study are summarized in Table 1.

**Table 1:** Strains used in this study

Species	*n*
*S.uberis*	53
*S. dysgalactiae*	41
*S. agalactiae*	27
Other *Streptococci*	14

### DNA isolation

Bacterial DNA was extracted using the ExtractMe DNA bacteria kit (Blirt, Gdańsk, Poland) according to the manufacturer’s protocols. Eluted DNA concentrations were measured using the BioSpectrometer® (Eppendorf, Hamburg, Germany) and stored at −20 °C for further analysis.

### Detection of virulence genes of *S. agalactiae, S. uberis* and *S. dysgalactiae*

Virulence genes for *S. uberis* (*sua, pauA/skc, gap C, has C, lbp, cfu, has B, has A)*, *S. agalactiae (pava, scpB, rib, bac, cylE, bca, cfb, lmb)* and *S. dysgalactiae (eno, napr)* were amplified using PCR. The confirmation of *S. agalactiae, S. uberis and S. dysgalactiae*, has been previously described [14]*.* Primer sequences, product sizes and annealing temperatures are summarized in Table 2. All primers were synthesized by Genomed S.A. (Warsaw, Poland). Amplification reactions were carried out with HotStarTaq Plus Master Mix Kit (Qiagen, Hilden, Germany) in a nexus gradient thermocycler (Eppendorf, Hamburg, Germany) using the same PCR protocol as described before [14]. All PCR reactions were carried out with positive and negative controls. Some PCR products of interest were randomly chosen and purified using a CleanUp kit (A&A Biotechnology, Gdynia, Poland) for sequencing (Genomed, Warsaw, Poland) to confirm the specificity of the obtained amplicons.

**Table 2:** Primer sets used for the detection of virulence genes in *Streptococcus spp*.

Primer function	Putative function	Target gene	Primer sequence (5′–3′)	Amplicon size (bp)	Annealing temperature (°C)	Reference
Virulence genes						
*S.uberis*						
Adhesion molecue-	Adherence and invasion of mammary epithelial cells	*sua*	TCAACTTGACGAATCGCTTG TCAGCCATTGTTTCTGCTTG	480	47	[20]
Plasminogen activator	colonization	*pauA/skc*	*CGGGTTGAAGAACCTATCACTC* *TGGAAGTTGACCAAGAGAATTG*	255	50	[20]
Surfacedehydrogenase protein	colonization	*gapC*	GCTCCTGGTGGAGATGATGT GTCACCAGTGTAAGCGTGGA	200	51	[11]
CAMP factor	Forms pores in host-cell membran	*cfu*	*TATCCCGATTTGCAGCCTAC* *CCTGGTCAACTTGTGCAACTG*	205	50	[11]
Lactoferrin binding protein	Binding Lactoferrin	*lbp*	CGACCCTTCAGATTGGATTC TAGCAGCATCACGTTCTTCG	698	50	[11]
Hyaluronic acid capsule	Resistance to phagocytosis	*hasA*	*GAAAGGTCTGATGCTGATG* *TCATCCCCTATGCTTACAG*	319	44	[33]
		*hasB*	*TCTAGACGCCGATCAAGC* *TGAATTCCTATGCGTCGATC*	532	47	[33]
		*hasC*	T*GCTTGGTGACGATTTGATG* *GTCCAATGATAGCAAGGTCAC*	225	47	[33]
S.agalactiae						
Adherence and virulence protein A	Binding to immobilized fibronectin	*pavA*	*TTCCCATGATTTCAACAACAAG* *AACCTTTTGACCATGAATTGGTA*	495	47	[20]
Streptococcal C5a peptidase- adhesion	Prevents neutropil, promotes adherence	*scpB*	*AGTTGCTTCTTACAGCCCAGA* *GGCGCAGACATACTAGTTCCA*	567	51	[20]
Surface protein Rib-	Resistance to proteases	*rib*	CAGGAAGTGCTGTTACGTTAAAC CGTCCCATTTAGGGTCTTCC	369	51	[34]
C beta protein	Binds to the immuno-globulin A	*bac*	*CTATTTTTGATATTGACAATGCAA* *GTCGTTACTTCCTTGAGATGTAAC*	592	46	[35]
Β-haemolisin	Promotes invasion of host cells	*cylE*	*TGACATTTACAAGTGACGAAG* *TTGCCAGGAGGAGAATAGGA*	248	47	[36]
C alfa protein	Adherence to epithelial cells	*bca*	TAACAGTTATGATACTTCACAGAC ACGACTTTCTTCCGTCCACTTAGG	535	49	[37]
CAMP factor	Forms pores in host-cell membran	*cfb*	ATGGGATTTGGGATAACTAAGCTAG AGCGTGTATTCCAGATTTCCTTAT	193	50	[38]
Lamining-binding protein	Promote adherence to host laminin	*lmb*	AGTCAGCAAACCCCAAACAG GCTTCCTCACCAGCTAAAACG	397	50	[39]
S.dysgalactiae						
α-enolase	Plasminogen binding	*eno*	ATGTCAATTATTACTGATGT CTATTTTTTTAAGTTATAGA	1308	36	[17]
Nephritis-associated plasminogen-binding receptor	Plasminogen binding	*napr*	GTTAAAGTTGGTATTAACGGT TTGAGCAGTGTAAGACATTTC	963	45	[17]

### Biofilm assay

The experiment was performed using polystyrene microtiter plates with flat bottoms based on the techniques described by Ebrahimi et al. [13]. All isolates were grown in tryptic soy broth (TSB, Oxoid, Great Britain) at 37 °C for 16 h. The bacterial cells were centrifuged at 6000 g for 10 min. Supernatants were removed and the cell pellets were resuspended in 5 mL of fresh medium (TSB) to achieve an absorbance of 1.00 at 595 nm. The optical density (ODs) of the bacterial suspensions were measured using the BioSpectrometer® (Eppendorf, Hamburg, Germany). The suspensions were then diluted 1:40 in fresh TSB, and 200 μl of the diluted cell suspension was added to each well of the microtiter plate (8 wells in a single row for one strain). Plates were incubated at 37 °C for 24 h. The plates were then washed three times with sterile phosphate-buffered saline (PBS). The inverted plates were dried at ambient temperature for 1 h. Next, the plates were stained with 200 μl of 0.2% aqueous crystal violet solution for 15 min. The plates were then washed three times with sterile PBS to remove excess dye. Crystal violet bound to the biofilms was extracted with 200 μl of an 80:20 (*v*/v) mixture of ethyl alcohol and acetone and the absorbance was measured at 595 nm using an ELISA plate reader (Sunrise absorbance reader, Tecan, Austria). Non-inoculated TSB wells stained with crystal violet were used as negative controls. All assays were performed in triplicate with 8 replicates for each strain. Interpretation of biofilm production was according to the criteria described by [15]. The mean optical density (OD) of the negative control +3 standard deviations of negative control was considered the cut-off (ODc = 0,2), and the biofilm producers were therefore categorized as follows:

- not a biofilm producer: OD ≤ ODc, (all strains which OD values were below 0,2)
- weak biofilm producer ODc: < OD ≤ 2 × ODc, (all strains which OD values were above 0,2 and below 0,4)
- moderate biofilm producer: 2 × ODc < OD ≤ 4 × ODc (all strains which OD values were above 0,4 and below 0,8)
- strong biofilm producer: OD > 4 × ODc. (all strains which OD values were above 0,8).

### Molecular serotyping of *Streptococcus agalactiae*

The assay was performed by multiplex PCR using a protocol previously described by Poyart el al. [16]. The protocol is based on two multiplex PCRs, one containing the primer pairs specific for serotypes Ia, Ib, II, III, and IV and the other containing primer pairs specific for serotypes V, VI, VII, VIII. Serotype IX was not investigated using this method. Non-typeable isolates were designated ‘NT’. To confirm the specificity of the obtained amplicons, some PCR products of interest were randomly chosen and purified using a CleanUp kit (A&A Biotechnology, Gdynia, Poland) for sequencing (Genomed, Warsaw, Poland).

## Results

### Biofilm production

Most of the analysed strains, which were classified into four groups (*S. agalactiae, S. uberis, S. dysgalactiae* and other streptococci), showed the ability to produce biofilm (over 70%). The occurrence of strains characterized as strong biofilm producers was noted in only two species (*S. uberis* and *S. agalactiae*; 6% and 7.5%, respectively) [Table 3].

**Table 3:** Biofilm production capability and formation intensity

Species	No biofilm producers	Weak biofilm producers	Moderate biofilm produres	Strong biofilm producers
*S.uberis*	12 (23%)	33 (62%)	5 (9%)	3 (6%)
*S.dysgalactiae*	5 (13%)	26 (63%)	10 (24%)	0 (0%)
*S.agalactiae*	2 (7,5%)	20 (74%)	3 (11%)	2 (7,5%)
other	3 (21%)	10 (72%)	1 (7%)	0 (0%)

*S. agalactiae* (*n* = 27), *S. uberis* (*n* = 53), *S. dysgalactiae* (*n* = 41) and other streptococci (*n* = 14)

### Detection of virulence genes

Virulence genes were searched for in the three species of streptococci most frequently appearing in our study, i.e., *S. agalactiae, S. uberis* and *S. dysgalactiae*. For the remaining category of other streptococci (*n* = 14), invasiveness genes were not determined due to species diversity in this group (four different species).

Regarding *S. agalactiae* (*n* = 27), the eight most common virulence genes in this species were selected for analysis. Among the analysed isolates, the presence of only four genes was confirmed: *rib* (33%), *cylE* (78%), *bca* (37%), *cfb* (100%). The genes *pavA*, *scpB*, *bac* and l*mb* did not occur in any of the tested strains [Table 4].

**Table 4:** Frequency of occurrence [no. (%)] of the chosen virulence genes

*S.agalactiae*								
	*pavA*	*scpB*	*rib*	*bac*	*cylE*	*bca*	*cfb*	*lmb*
*n*	0	0	9	0	21	10	27	0
%	0	0	33	0	78	37	100	0
*S. uberis*								
	*sua*	*pauA/skc*	*gapC*	*cfu*	*lbp*	*hasA*	*hasB*	*hasC*
*n*	51	50	52	10	0	9	42	44
%	96	94	98	19	0	17	79	83
*S.dysgalactiae*								
	*eno*	*naprl*						
*n*	31	34						
%	76	83						

*Streptococcus uberis* (*n* = 53), *Streptococcus agalactiae* (*n* = 27), *Streptococcus dysgalactiae* (*n* = 41), and other *Streptococcus* species (*n* = 14)

As for the strains of *S. uberis* (*n* = 53), eight genes connected with virulence were selected for analysis. Only one gene, *lbp*, was not found in the isolates of this species. The other genes were quite abundant at70%. One exception was a gene of the has operon, *hasA*, which was identified in only 17% of the analysed strains [Table 4].

The last group submitted to analysis consisted of isolates of *S. dysgalactiae* (*n* = 41). In this group, only two genes, *eno* and *napr*, were selected for the tests, mainly because there is very little knowledge about the mechanisms of invasiveness in this species [17] Regarding our isolates, the presence of these two genes was confirmed in 76% and 86% of strains, respectively [Table 4].

### Molecular serotyping of *Streptococcus agalactiae* and associated virulence genes

The presence of serotypes Ia, Ib, II, III, IV and V was confirmed among the analysed strains, and the most numerous group consisted of serotypes Ia and II (*n* = 8). In our experiment, there were 6 strains which could not be classified into any of the analysed serotypes (NT). In addition, an analysis was performed regarding the presence of virulence genes in the individual serotypes. In the case of serotype Ia, only one profile was observed: *rib, cylE, cfb*. The profile *cylE, bca, cfb* dominated in the second most numerous group, i.e., serotype II. In the NT strains, there was only one gene: *cfb* [Table 5].

**Table 5:** Results of molecular serotyping of *S. agalactiae* strains (*n* = 27) (including virulence profiles)

Serotype	*n*	Profile			
		*rib, cylE, cfb,*	*cylE, bca, cfb*	*cylE, cfb*	*cfb*
Ia	8	8	0	0	0
Ib	2	2	0	0	0
II	8	0	7	1	0
III	1	0	1	0	0
IV	1	0	0	1	0
V	1	0	1	0	0
NT	6	0	1	0	5

## Discussion

The presence or absence of virulence factors in pathogenic aetiological agent of a disease is an important variable that decides the course of the illness itself. The enzymes and toxins produced by bacteria or the ability of bacteria to produce biofilm help microorganisms survive in infected tissues either through direct impact on host stromal cells or by affecting host defence mechanisms [18]. In our experiment, most of the analysed isolates of *Streptococcus* spp*.* demonstrated an ability to produce biofilm (over 70%), many of which represented strains forming a weak structure. Such high abundance in this group can be explained by the fact that determinations in strains were made after a 24-h incubation period. Moliva et al. [19] recently showed that strains of *S. uberis* begin to form biofilm structure as early as after 2 h of incubation, but mature biofilm is not formed until after 48 h. It is possible that the relatively longer incubation of isolates in our study influenced the results. Nevertheless, we are convinced that the high occurrence of *Streptococcus* spp*.* strains able to produce biofilm in the geographical area we investigated presents a serious challenge for both dairy farmers and veterinary doctors. As mentioned earlier, the production of this specific structure by bacteria can have a considerable impact upon treatment efficiency. From a clinical point of view, there is a significant relationship between the ability to create bacterial biofilm and antibiotic treatment [7]. Bacteria that form a ‘biological membrane’ can be characterized by up to 1000-fold lower sensitivity to the effects of some active substances compared to strains growing in planktonic form. In addition, some antibiotics, for example tetracyclines or erythromycin, can stimulate the formation of biofilm. This was observed in the case of *Staphylococcus epidermidis*, where following the administration of these antibiotics there was an observed increase in the expression of the gene responsible for the formation of biofilm (*ica*) [7]. In our earlier studies conducted on the same strains [14], we found a high level of resistance of these pathogens to tetracycline. This observation may indicate that the said active substance is overused in the analysed area, and simultaneously may explain the presence of such a high number of strains able to produce biofilm. However, we have not observed any correlation between biofilm formation ability and other virulence factors occurrence. This may provide that biofilm formation is not essential in the pathogenesis of mastitis in dairy cattle in the analysed area for now.

Next, we analysed bacteria searching for the presence of the selected virulence genes. Eight genes were selected or *S. agalactiae*, of which four (*rib, cylE, bca, cfb)* were determined to be present in the analysed strains. These results were unsurprising because their presence had been previously confirmed in bovine strains in countries such as Brazil, India or Egypt, and the frequency of occurrence of these genes is local in character [12, 19–21]*.* The situation differs with respect to the genes *scpB, lmb, pavA* and *bac,* whose presence was not detected in the strains we analysed. On the one hand, these genes are mainly associated with human strains, and therefore their absence is not puzzling [12, 20]. On the other hand, the genes *scpB, pavA* or *lmb* have been detected in isolates of *S. agalactiae* derived from cattle with mastitis in Asia and Africa [20–22]. Thus, our findings indicate that these four genes probably do not play important roles in the pathogenesis of udder inflammation in dairy cattle in the region, which may be connected with the different conditions and management practices for dairy cattle in Europe.

In the case of *S. agalactiae*, we performed additional molecular serotyping of the isolated strains. Such investigations are extremely important considering both epidemiology and prophylaxis, as they may aid the development of multivalent vaccines containing capsular polysaccharides [23]. Our isolates belonged to serotypes from Ia to V. The occurrence of these serotypes need not be surprising, as they were isolated previously in the United States and Europe; however, the dominance of serotype Ia in our study is quite puzzling because according to Delannoy et al. [24], this serotype is mostly present in humans. Serotype III is usually dominant in cattle but in our experiment it was confirmed in only one case. Nevertheless, the presence of serotype Ia in cattle has also been confirmed in the eastern part of China, Germany and the USA [23, 25, 26]. These observations collectively seem to support the claim that the presence of specific serotypes may be a local characteristic due to several factors (e.g., breed of cattle, geographic location, farm management), which highlights the importance of serotyping studies for selecting appropriate prevention measures [23, 25, 26]. In our study, we also found strains not classified to any serotype. This was not surprising because such strains are quite frequently observed among *S. agalactiae* isolated from cattle [25]. Nonetheless, we did not determine serotype IX in our experiment, and consequently, the number of strains in the NT group may be unduly high. In addition, we analysed of the presence of virulence genes in individual serotypes of *S. agalactiae*. Interestingly, despite the frequent presence of serotype IA that is typical of human diseases, we did not detect in this serotype genes characteristic for human strains of *S. agalactiae* (*scpB, lmb, pavA* or *bac*). In the two most often isolated serotypes IA and II, the dominance of two virulence profiles was observed: *rib, cylE, cfb,* and *bca, cylE, cfb*, respectively*.* These serotypes differed from each other by the presence of two genes: *rib* and *bca.* These genes belong to the same family, the alpha-like protein (Alp) family, and play important roles in the pathogenesis of illnesses caused by these bacteria, and they are also acknowledged as vaccine candidates [23]. Our results suggest that vaccines should contain at least two genes from this family because their presence may be correlated with the dominant serotype. However, there is a need to kept in mind that in the case of these bacteria there is a possibility of horizontal or lateral gene transfer. According to the Richards et al. [27] *S.agalactiae* can transfer genetic material both to their own species and other mastitis pathogens such as *S.uberis* or *S.dysgalactiae,* which is one of the mechanisms of these bacteria for adaptation to the bovine environment. However, our study was performed on a relatively small number of strains isolated from just one species, and further studies in this direction are necessary.

Eight genes (*sua, pauA/skc, gapC, cfu, lbp, hasA, hasB, hasC*) were selected for *S. uberis*, of which the following were most frequent: *gapC* (98%), *sua* (96%) and *pauA/skc (*94%). The presence of the gene *lbp* was not detected among the analysed strains. The three genes dominant in our study were previously confirmed at similar levels in Argentina, India, Great Britain, New Zealand and the USA [11, 28–31]. These genes are considered as good targets for vaccine development due to frequent occurrence and their highly conserved nature at the nucleotide and amino acid levels [11, 28, 32]. In our experiment, we also chose to analyse genes of the has operon (*hasABC*), which is responsible for capsule production. The key role in this process is played by the gene *hasA*, which was rather rare (17%) among the analysed strains. This finding suggests that in the region investigated, the ability of *S. uberis* to produce capsules does not play a key role in mastitis [11].

In *S. dysgalactiae*, we focused on two genes that encode proteins responsible for binding host plasminogen (*naprl* and *eno*). Both occurred quite frequently among the analysed strains (approximately 80%). Similar results for these two genes have been observed among *S. dysgalactiae* strains isolated from fish and pigs in Asian countries [17].

## Conclusions

In summary, the experiment reported above showed that strains of *Streptococcus* spp. in northeastern Poland possess several invasiveness factors, which can considerably influence the course of disease and treatment. The data obtained during our study constitute a preliminary step towards further research targeting a better understanding of the dominant virulence mechanisms among these bacteria, and they may aid the development of target prevention programmes for this region of the country.
